# Humeral Head Preservation after Neglected Glenohumeral Dislocation by Latarjet and Infraspinatus Remplissage—A Case Report

**DOI:** 10.3390/jcm13164862

**Published:** 2024-08-17

**Authors:** Pieter van Gerven, Nikki Buijs, Leanne Blaas, J. Zhang Yuan, Jacobus A. de Priester, Robert Jan Derksen

**Affiliations:** 1 Department of Surgery, Zaandam Medical Center, Koningin Julianaplein 58, 1502 DV Zaandam, The Netherlandsderksen.r@zaansmc.nl (R.J.D.); 2 Department of Radiology, Zaandam Medical Center, Koningin Julianaplein 58, 1502 DV Zaandam, The Netherlands

**Keywords:** glenohumeral dislocation, Latarjet procedure, infraspinatus remplissage, off-track Hill-Sachs deformity, bony Bankart lesion, shoulder arthroplasty

## Abstract

**Background:** Neglected anterior glenohumeral dislocations provide a challenging problem for physicians. For many patients with these injuries, reverse shoulder arthroplasty has been the treatment of choice, although the preservation of the patient’s own humeral head might have significant advantages. **Methods:** We present a case of a 66-year-old male with a neglected anterior glenohumeral dislocation that he sustained 6 weeks prior when he was hit by a car as a pedestrian. Radiographic imaging revealed a large off-track Hill-Sachs deformity and a fracture of the greater tuberosity in addition to the persisting glenohumeral dislocation. We performed open reduction and to aid stability, an infraspinatus tendon remplissage and a Latarjet procedure were performed. **Results:** Apart from minor and self-limiting neuropraxia, recovery was without complications. At 24 month follow-up, the patient had no impairment in general activities, had no residual pain, and had a good active range of motion. **Conclusions:** The authors, therefore, believe that a combination of infraspinatus tendon remplissage and the Latarjet procedure seems a feasible alternative for reverse shoulder arthroplasty and can preserve the patient’s own humeral head.

## 1. Introduction

The Latarjet procedure is commonly used to stabilize chronic anterior shoulder instability or recurring glenohumeral dislocations [1,2,3,4,5]. It consists of a coracoid osteotomy and the fixation of the coracoid process on the anterior glenoid rim and aids glenohumeral stability. A case report describing this technique after the fracture/dislocation of the humeral head in a young male has been published recently by our study group [6] and has been replicated by another institution for a similar case of neglected glenohumeral dislocation [7]. Remplissage using the infraspinatus tendon is another procedure commonly performed in recurring anterior glenohumeral dislocations, especially when an extensive off-track Hill-Sachs deformity is present [8,9]. 

This report describes the combination of a Latarjet procedure and the remplissage of the infraspinatus tendon for a novel indication: a neglected anterior glenohumeral dislocation with a severe off-track Hill-Sachs deformity and a fracture of the greater tuberosity. The Latarjet procedure serves a dual purpose in this case, both facilitating the reduction of the persisting glenohumeral dislocation, as well as stabilizing the glenohumeral joint to prevent recurrence. The infraspinatus remplissage further adds to shoulder stability as well as filling the extensive Hill-Sachs deformity. 

In these challenging cases of delayed detection of a glenohumeral dislocation, usually, a reverse shoulder arthroplasty is the procedure of choice. It is generally considered unfeasible to salvage the humeral head since the head, tendons, capsule, and labrum are considered to have been displaced for too long. However, by using both a direct Latarjet as well as an infraspinatus remplissage, these complicating factors can all be addressed. This combination has been used in the cases of recurring glenohumeral dislocations. However, to our knowledge, this is the first report in the literature of the combined use of these procedures for this specific indication of (sub)-acute and neglected glenohumeral dislocation. By combining the Latarjet and remplissage procedures for the case at hand, we aimed to show that in selected cases, a humeral head-preserving treatment is possible in a neglected (chronic) anterior glenohumeral dislocation (as opposed to the adage that a reverse shoulder arthroplasty should always be chosen in these cases).

## 2. Materials and Methods

### 2.1. Patient Information, Clinical Findings, and Diagnostic Assessment

This report describes the case of a 66-year-old male, who was referred to our outpatient clinic by his general practitioner with decreased function of the left shoulder and persistent pain after he was hit by a car whilst crossing the street as a pedestrian 6 weeks earlier. The patient was examined directly after the accident by the on-call general practitioner. He was misdiagnosed as merely having sustained a contusion, and at this time, no radiographs of the shoulder were made. 

His medical history included the extirpation of a cerebellopontine meningioma in 2008, radiation therapy for a local recurrence of this meningioma in 2020, hypertension, hypercholesterolemia, and mild obesity. At the outpatient clinic, we were consulted by the patient with a painful left shoulder and a severe decrease in range of motion (ROM). There was no neurological deficit in the affected arm. 

**Figure 1:**
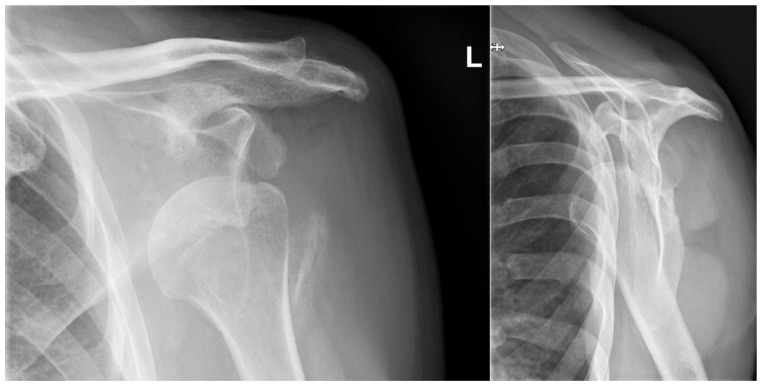
Preoperative radiographs. Anteroposterior and lateral “Y-view” radiographs showing the anterior glenohumeral dislocation of the affected shoulder.

**Figure 2:**
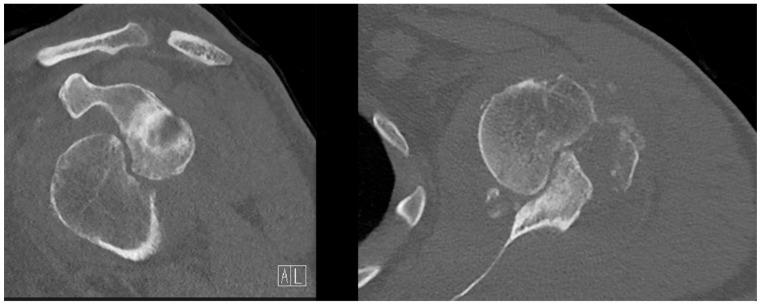
Preoperative CT scan. Transversal (**right**) and coronal (**left**) frames of the affected shoulder showing the large Hill-Sachs deformity. On the coronal image, the Bankart lesion on the anterior glenoid rim is clearly visible.

**Figure 3:**
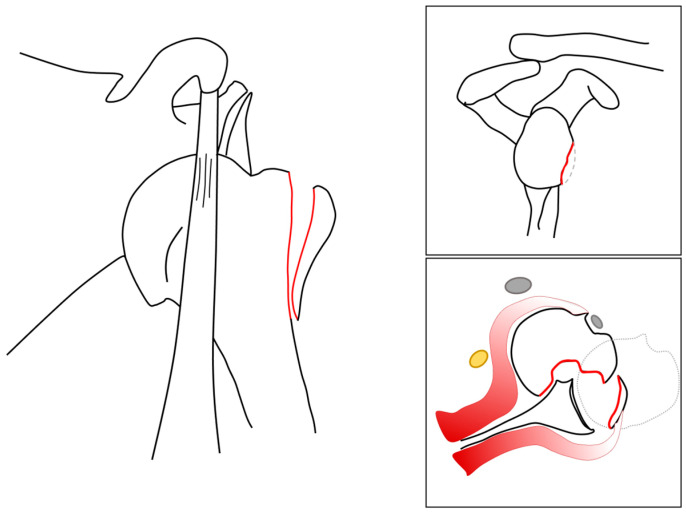
Preoperative situation. A graphic demonstration of the initial injury sustained by the patient. Note that there is both an anterior glenohumeral dislocation with concomitant severe Hill-Sachs deformity, a bony Bankart lesion, and a fracture of the greater tuberosity. The yellow structure depicts the axillary nerve, and the gray structures represent the conjoint tendon and long biceps tendon.

A radiographic assessment revealed an anterior dislocation in the glenohumeral joint, with a concomitant fracture of the greater tuberosity and a Hill-Sachs deformity as shown in Figure 1. The CT scan revealed a bony Bankart lesion of the anterior glenoid rim, and characterized the Hill-Sachs deformity to be large and off-track as shown in Figure 2. Due to the prolonged engagement with the glenoid rim during the 6 weeks that the head was dislocated, a substantial defect was mulled out. A graphic representation of this injury is presented in Figure 3. The patient was scheduled for surgery to be performed in the oncoming days.

### 2.2. Therapeutic Intervention

The patient was in beach chair position, and the procedure was performed under general anesthesia combined with an interscalene plexus blockade. We performed a deltopectoral approach of the shoulder. A coracoid osteotomy was performed to gain access to the humeral head, which had protruded anteriorly and medially into the deltopectoral axillary groove. Using a bone elevator, and by performing longitudinal traction and external rotation on the affected arm, we were able to reduce the humeral head to its anatomic position in the glenoid fossa. 

After the glenohumeral dislocation was reduced, the enlarged Hill-Sachs lesion was deemed too large to make do with Latarjet alone. In order to aid stability, and to address the large off-track Hill-Sachs deformity, a remplissage using the infraspinatus tendon was performed. Due to the fractured greater tuberosity, the Hill-Sachs deformity could be reached easily via the deltopectoral approach and a suture anchor (Twinfix 4.5 mm, Smith & Nephew©, London, UK) was placed in the deformity. The sutures were positioned in the infraspinatus tendon, but not yet tightened.

After this, the fractured greater tuberosity was reduced, and fixed using two cannulated headless compression screws (4.0 mm Synthes©, West Chester, PA, USA). We then tightened the sutures through the infraspinatus tendon to complete the remplissage. Finally, the coracoid tip with the attached tendons (m. coracobrachialis, m. pectoralis minor, and the short head of the m. biceps brachii) that was osteotomized in the first step was transferred through the tendon of the m. subscapularis, and was fixed to the anterior rim of the glenoid fossa with two headless compression screws (3.0 mm Synthes©).

**Figure 4:**
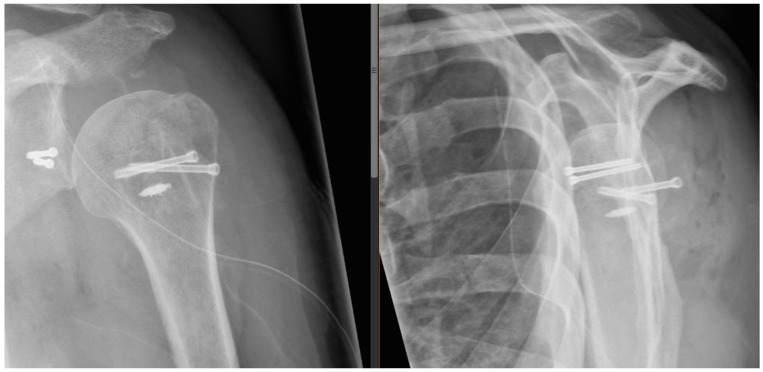
Postoperative radiographs. Anteroposterior and lateral “Y-view” radiographs showing the postoperative situation.

**Figure 5:**
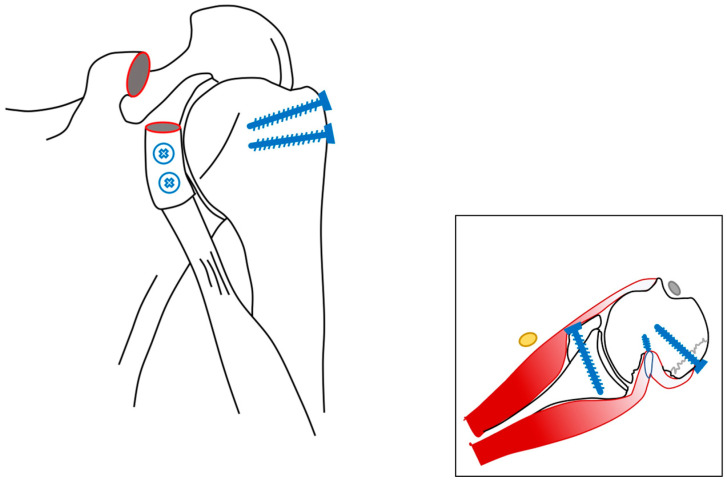
Postoperative situation. A graphic demonstration of the postoperative situation. Note that the tip of the coracoid bone has been transferred to the anterior rim of the glenoid (Latarjet procedure), that the larger tuberosity fracture has been reduced and fixated, and that a remplissage of the infraspinatus tendon has been performed to aid in stability, as well as to fill the Hill-Sachs deformity.

Postoperative radiographs and CT scans obtained one day postoperatively showed good positioning of all the bony fragments, and no sign of complications as shown in Figure 4. A graphic representation of the postoperative situation is presented in Figure 5.

Postoperatively, intravenous antibiotics were administered for a period of 24 h. A single Redon drain was left in situ during these initial 24 h, and was removed on the surgical ward. Discharge from the hospital occurred on postoperative day 2. The affected shoulder was immobilized using a Gilchrist sling for 7 days, after which active mobilization under the supervision of a specialized physiotherapist was started.

## 3. Follow-Up and Outcomes

At the first postoperative outpatient clinic visit one week after surgery, the patient was doing well, and the pain was adequately managed. He started physiotherapy consultations twice a week. For 6 weeks, forward flexion and abduction were allowed as far as 90 degrees, or less than that if pain restricted the patient.

At the second outpatient clinic visit one month after the procedure, the patient complained of a tingling sensation in the medial forearm. An EMG examination at the neurology department revealed a mild plexopathy with signs of advanced reinnervation.

At the third outpatient clinic visit 3 months after the surgery was performed, the patient reported an improvement in both his pain scores, as well as range of motion. During the physical examination, the abduction was 45 degrees, and forward flexion was 20 degrees. Rotation was, however, still impaired significantly, with a maximum external rotation of 5 degrees. Nearly all of the neurological symptoms that the patient experienced at the previous outpatient clinic visit had resolved. Radiographic examination showed a good position of both the fixated greater tuberosity, as well as of the coracoid bone graft on the anterior glenoid rim. The patient was advised to continue with his physiotherapy consultations.

At the 8-month follow-up, the patient was doing well. He reported no residual pain and was unimpaired in the general daily activities. At 12 months, his active ROM comprised of 90 degrees of abduction, 90 degrees of forward flexion, and 15 degrees of external rotation. Follow-up radiographs show anatomical reduction and no signs of complications.

**Figure 6:**
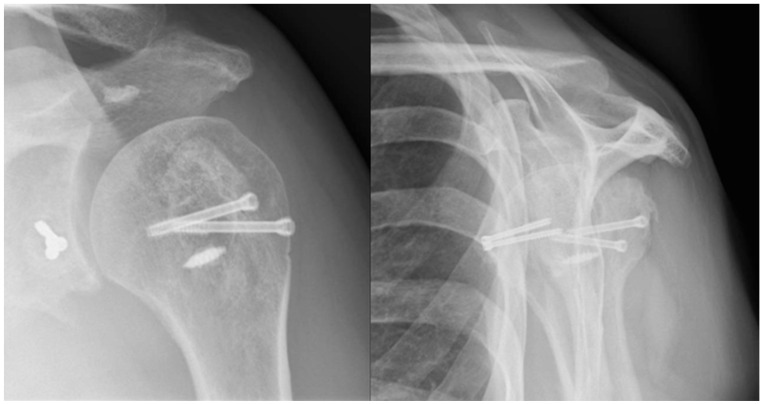
Follow-up radiographs. Anteroposterior and lateral “Y-view” radiographs at 24 months showing no change in the reduction, and no signs of complications.

**Figure 7:**
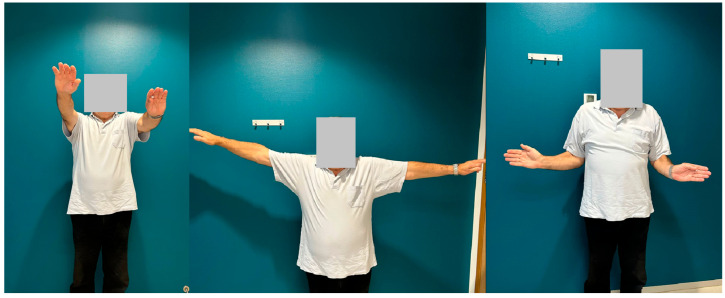
Clinical range of motion. A representation of the patient’s active range of motion in forward flexion, abduction, and external rotation at the 24-month follow-up.

At the final follow-up visit 24 months after the surgery, the patient had no pain and was satisfied with his function. Radiographs showed no differences compared to the previous radiographs and showed no complications as shown in Figure 6. The active ROM was measured and showed a forward flexion of 90°, extension 42°, abduction 98°, and an external rotation of 10° as shown in Figure 7. Three questionnaires were assessed. The Constant Shoulder Score (CSS) of the dislocated arm was 76. The CSS of the unaffected arm was 24. The Oxford Shoulder Score was 35 and the Quick Disabilities of the Arm, Shoulder, and Hand (Quick DASH) was 22.5.

## 4. Discussion

Neglected and chronic anterior glenohumeral dislocations provide a challenging problem for physicians for several reasons. First, the safe retrieval of the humeral head from the deltopectoral axillary groove after a neglected anterior glenohumeral dislocation may be challenging because of capsular fibrosis, retraction, and the location of the humeral head in relation to the brachial plexus [6]. Second, often there is a large posterolateral bony defect (mulled out Hill-Sachs deformity) resulting from prolonged friction between the humeral head and the anterior rim of the glenoid. Also, the anterocaudal rim of the glenoid is usually blunted from the grinding motions of the head, or fractured during the dislocation.

This combination of the aggravated Hill-Sachs deformity and Bankart lesion, as well as the capsular fibrosis and retraction, makes this injury hard to resolve and commonly, physicians will resort to arthroplasty [10,11,12]. However, especially in younger patients, a treatment option that preserves the patient’s own humeral head has some obvious advantages [7,13,14,15]. A head-preserving treatment better preserves the patient’s proprioception, is less costly, and has a lesser risk of prosthesis-related infection. Also, revision surgery might be necessary for arthroplasty, and there is a lesser risk after successful head-preserving treatment. Finally, because the center of rotation of a reverse shoulder arthroplasty lies further medially, rotational ROM could benefit from a head-preserving treatment.

In this particular case, since the rotator cuff tendons were intact and of good quality, and no preexistent omarthrosis was present, we opted for a head-preserving treatment option instead of arthroplasty. After a successful reduction of this unstable injury type, which after a marked delay in diagnosis inadvertently has to be performed open, additional measures are required to prevent early re-dislocation. Some surgeons perform temporary arthrodesis by using either screws or K-wires to perform glenohumeral or acromiohumeral arthrodesis [16,17]. This can be carried out either as an independent procedure, or following a soft tissue Bankart repair or a Latarjet procedure. Such a temporary arthrodesis of the shoulder, however, requires prolonged immobilization, and patients will, therefore, require even more extensive physiotherapy to regain their function and ROM.

There are several other options to improve shoulder stability following the reduction of glenohumeral dislocation. A recent study found that even when complicating factors such as a bony Bankart lesion or a Hill-Sachs deformity were not present, 30% of the patients suffered from an anterior or craniocaudal (sub) luxation after the open reduction of a neglected glenohumeral dislocation [18]. Filling the Hill-Sachs deformity using an infraspinatus tendon remplissage is one of the options to improve shoulder stability. This procedure, like the Latarjet procedure, is typically utilized in patients with severe chronic shoulder instability resulting from recurring glenohumeral dislocations [19]. Significantly less data are available on the use of either of these procedures in the primary treatment of a semi-acute and neglected glenohumeral dislocation.

In the literature, a study described four cases in which a remplissage procedure following an open reduction of longer-existing anterior glenohumeral dislocation was used to provide extra stability, and led to a good clinical outcome [20]. Capsulolabral Bankart repair either with or without bone grafting such as a Latarjet procedure can also be used as a standalone procedure for this indication [21,22].

The Latarjet procedure and the infraspinatus tendon remplissage are used in conjunction for specific indications, such as in patients with recurrent glenohumeral dislocations with a large Hill-Sachs deformity (>25%), an off-track localization, or anterior glenoid bone loss [23]. To our knowledge, our case is the first time the combination of a Latarjet procedure and an infraspinatus remplissage has been performed in a patient with a longer-existing and neglected primary anterior glenohumeral dislocation.

The complications of the Latarjet procedure range from 15% to 25%, and include (deep) surgical site infections, graft nonunion, graft fractures, or graft malunions and neurological deficits [4,24,25]. The infraspinatus remplissage is reported to have less complications, especially if performed when the humeral head is reduced open so no additional surgical approach of the shoulder is necessary [25]. Apart from a minor self-limiting neurological deficit, the patient did not develop any of the complications associated with either the open reduction, the Latarjet procedure, or the infraspinatus remplissage. The functional outcome was above expectations, and the patient was satisfied with the treatment results.

## 5. Conclusions

In certain cases of the delayed detection of a glenohumeral dislocation with added complicating factors, it seems feasible to preserve the humeral head using this combination of Latarjet procedure and infraspinatus remplissage. Obviously, this constitutes only one successful case, and firmer inferences on this procedure and the impact on the quality of life for patients suffering from this type of injury can only be made after more successful cases and cohort analysis.

## Data Availability

The original contributions presented in the study are included in the article, further inquiries can be directed to the corresponding authors.
